# Whole-Genome Profile of Greek Patients with Teratozοοspermia: Identification of Candidate Variants and Genes

**DOI:** 10.3390/genes13091606

**Published:** 2022-09-08

**Authors:** Maria-Anna Kyrgiafini, Themistoklis Giannoulis, Alexia Chatziparasidou, Nikolaos Christoforidis, Zissis Mamuris

**Affiliations:** 1 Laboratory of Genetics, Comparative and Evolutionary Biology, Department of Biochemistry and Biotechnology, University of Thessaly, Viopolis, Mezourlo, 41500 Larissa, Greece; 2 Laboratory of Biology, Genetics and Bioinformatics, Department of Animal Sciences, University of Thessaly, Gaiopolis, 41336 Larissa, Greece; 3 Embryolab IVF Unit, St. 173-175 Ethnikis Antistaseos, Kalamaria, 55134 Thessaloniki, Greece

**Keywords:** teratozoospermia, male infertility, whole-genome sequencing (WGS), next-generation sequencing, Greece

## Abstract

Male infertility is a global health problem that affects a large number of couples worldwide. It can be categorized into specific subtypes, including teratozoospermia. The present study aimed to identify new variants associated with teratozoospermia in the Greek population and to explore the role of genes on which these were identified. For this reason, whole-genome sequencing (WGS) was performed on normozoospermic and teratozoospermic individuals, and after selecting only variants found in teratozoospermic men, these were further prioritized using a wide range of tools, functional and predictive algorithms, etc. An average of 600,000 variants were identified, and of them, 61 were characterized as high impact and 153 as moderate impact. Many of these are mapped in genes previously associated with male infertility, yet others are related for the first time to teratozoospermia. Furthermore, pathway enrichment analysis and Gene ontology (GO) analyses revealed the important role of the extracellular matrix in teratozoospermia. Therefore, the present study confirms the contribution of genes studied in the past to male infertility and sheds light on new molecular mechanisms by providing a list of variants and candidate genes associated with teratozoospermia in the Greek population.

## 1. Introduction

Infertility is defined by the *World Health Organization* (WHO) as the failure to conceive after at least 12 months of regular and unprotected sexual intercourse [1]. It is considered a major health problem that affects the couple’s psychology and social life [2,3], but at the same time, it causes a significant economic burden on the health care system and patients [4]. Moreover, it is estimated that more than 186 million people are affected worldwide [5], and in half of these cases, after a thorough examination, a male cause seems to be present alone or in conjunction with female causes [6]. Based on semen analysis and defects associated with sperm quality or quantity, several subtypes of male infertility can be defined, such as asthenozoospermia, oligozoospermia, or teratozoospermia [7]. Specifically, when in semen less than 4% of spermatozoa have normal morphology, according to WHO, the sample is characterized as teratozoospermic. 

Male infertility is considered a multifactorial disorder [8], and it is estimated that genetic factors are involved in 15% of cases and more [9]. Although extensive research has resulted in significant advances in the field, the identification of specific genes and mutations is a great challenge, as more than 2000 genes are required only for spermatogenesis, a crucial process for fertility [9,10]. Especially for particular subtypes of male infertility, such as teratozoospermia, detection of causal mutations that lead to specific defects in sperm parameters remains limited [8,10]. Furthermore, the fact that assisted reproductive technologies (ART) are being used more frequently than in the past proves the progress achieved in the area of male infertility, but it has been observed that ART outcomes differ between ethnic groups probably due to different genetic factors that contribute to infertility among populations [11,12]. However, the impact of ethnic differences on male infertility is not adequately addressed in research [12]. Today, whole-genome sequencing (WGS) enables the identification of common and even rare variants, which are often not captured at Genome-Wide Association Studies (GWAS) and SNP-chips [13], observed in specific geographical populations, which have an impact on phenotypic variation for a wide range of diseases in an accurate and cost-effective way [14,15]. 

Thus, this study aimed to perform WGS in teratozoospermic and normozoospermic individuals of the Greek population (a) to identify and further characterize variants, including rare variants, that can contribute to the pathogenic phenotype, and (b) to highlight genes potentially linked with teratozoospermia, a specific subtype of male infertility, and to explore their role. The ultimate goal of our study was to provide a valuable reference for future male infertility research, particularly regarding teratozoospermia. In this way, the detection of mutations contributing to the infertile phenotype in ethnic minorities may be of utmost importance for unraveling the genetic basis of male infertility by identifying new or rare variants and studying the genes on which they are found, and for improving the diagnosis of teratozoospermia, as well as the chances of successful ART. 

More specifically, with our study that is focused on the Greek population, as there are no other studies for the genetic background of teratozoospermia in the Balkans, we attempt to (a) provide a roadmap for future studies investigating different ART outcomes due to different genetic background among populations and maybe improve the chances for successful ART for Balkanian populations, and (b) provide preliminary information about genetic causes of male infertility specifically found on Balkan populations enabling comparison with other populations, e.g., Chinese, African, etc., in order to investigate the mechanisms causing teratozoospermia. 

## 2. Materials and Methods

### 2.1. Selection of Patients and Biological Material

For this study, human blood, as well as sperm samples, were collected from volunteers in cooperation with the “Embryolab IVF Unit” (55134 Thessaloniki, Greece) for the Spermogene research program. Ethical approval was obtained from the Ethics Committee, University of Thessaly (38221 Volos, Greece), and all individuals have given their approved written informed consent. 

All the volunteers recruited underwent an andrological examination and semen analysis was performed on samples derived from all of them. It should be noted that sperm samples were collected via masturbation after at least two to three days of abstinence from sexual intercourse. For semen analysis (seminogram), cell vision counting slides (Tek-Event) were used for cell counting and observation was conducted on Nikon Eclipse TS100, Nikon Eclipse E200, and Nikon Eclipse Ts2 microscopes. Semen analysis was performed according to WHO guidelines (fifth edition, 2010, https://apps.who.int/iris/handle/10665/44261 (accessed on 26 June 2022)) that include information on assessing semen volume, sperm count, motility, morphology, etc. These reference values proposed in this edition were used to define normozoospermic and teratozoospermic phenotypes.

Moreover, the inclusion criterion for this study was the Greek ethnicity as according to research, infertility exerts racial differences [16,17] and the ART outcomes differ between populations [11,12], but there is limited information regarding specific variations and polymorphisms associated with male infertility for ethnic groups found in the Balkans, and especially for the Greek population and teratozoospermia. Therefore, place of birth and relevant data were collected from the volunteers through the questionnaire which was required to fill in along with the consent form. Demographic information on the individuals enrolled in this study is presented in Table 1.

### 2.2. Sample Preparation and Whole-Genome Sequencing 

Genomic DNA was extracted from blood samples of teratozoospermic and normozoospermic individuals using the PureLink Genomic DNA Mini Kit (Invitrogen, Waltham, MA, USA—Catalog number: K182002) according to the manufacturer’s instructions. DNA quality and quantity were assessed by agarose gel electrophoresis and by Qubit 2.0 fluorometer using the Qubit dsDNA BR Assay Kit (Invitrogen, Waltham, MA, USA—Catalog number: Q32850), respectively. After that, three sequencing pools were created. More specifically, DNA extracted from ten normozoospermic individuals was used for the two pools (five individuals for each pool), and similarly, the third contained pooled DNAs from five teratozoospermic individuals. The DNAs were mixed equimolar for each pool in a final concentration of 100 ng/uL and a final quantity of 2 mg. 

Once their preparation was completed, DNA samples were shipped to Novogene (Cambridge, UK) where 100-bp paired-end libraries were constructed and sequenced using an Illumina HiSeq 3000 in a mean sequencing coverage of 30x. For the analysis of the FASTQ files produced, at first, the quality of the reads was evaluated using FASTQC (available online at: http://www.bioinformatics.babraham.ac.uk/projects/fastqc/, accessed on 26 June 2022) and then low-quality reads (minimum PHRED Score: 30), as well as adapter sequences, were discarded using Trimmomatic [18]. After quality control, the reads were aligned to a human reference genome (GRCh37/hg19) retrieved from the Ensembl database [19] using the Burrows-Wheeler aligner (BWA) [20]. Duplicate reads produced by polymerase chain reaction (PCR) were marked and removed by Picard tools before further analysis. SAM files of the alignment were then converted to BAM files using SAMtools [21] and individual BAM files for the two normozoospermic pools were merged to create one file representing normozoospermic individuals using SAMtools, too. Variant calling was performed using freeBayes [22] and as an output, the results were stored in variant call format (VCF). BCFtools [21] was then used to compare the VCF files from normozoospermic and teratozoospermic individuals to detect unique variants that are present only in one of the two groups, thus, they are not shared between normozoospermic and teratozoospermic individuals. Further analysis was performed for variants found only on men diagnosed with teratozoospermia as described earlier, as the aim was to identify variants and/or polymorphisms on teratozoospermic men that have the potential to contribute to the pathogenic phenotype and and may cause teratozoospermia in the Greek population. Moreover, as they exist only on patients, they could be used for the development of teratozoospermia biomarkers in the Greek population for effective diagnosis as well as for ART outcome improvement.

Following the detection of unique variants for teratozoospermic individuals, annotation was performed using the VEP tool (available at https://www.ensembl.org/Tools/VEP, accessed on 26 June 2022), provided by the Ensembl database. Furthermore, a list of databases, software tools, and additional prediction algorithms were used to retrieve more biological information and prevent bias in the filtering and prioritization of variants that were performed afterward, since it is common for potentially disease-relevant mutations to be ignored due to inadequate annotations [23]. Among them, the Single Nucleotide Polymorphism Database (dbSNP) [24], 1000 Genomes Project [25], and Genome Aggregation Database (gnomAD) [26] were used to obtain information about allele frequencies and the identification of rare/novel variants. Further analysis was carried out to predict the effect of variants on protein’s functionality or their potential pathogenic effect using Polymorphism Phenotyping v2 (PolyPhen2) [27], Sorting Intolerant From Tolerant (SIFT) [28], Combined Annotation Dependent Depletion (CADD) [29], and MutationAssessor [30].

### 2.3. Variant Prioritization 

**Figure 1:**
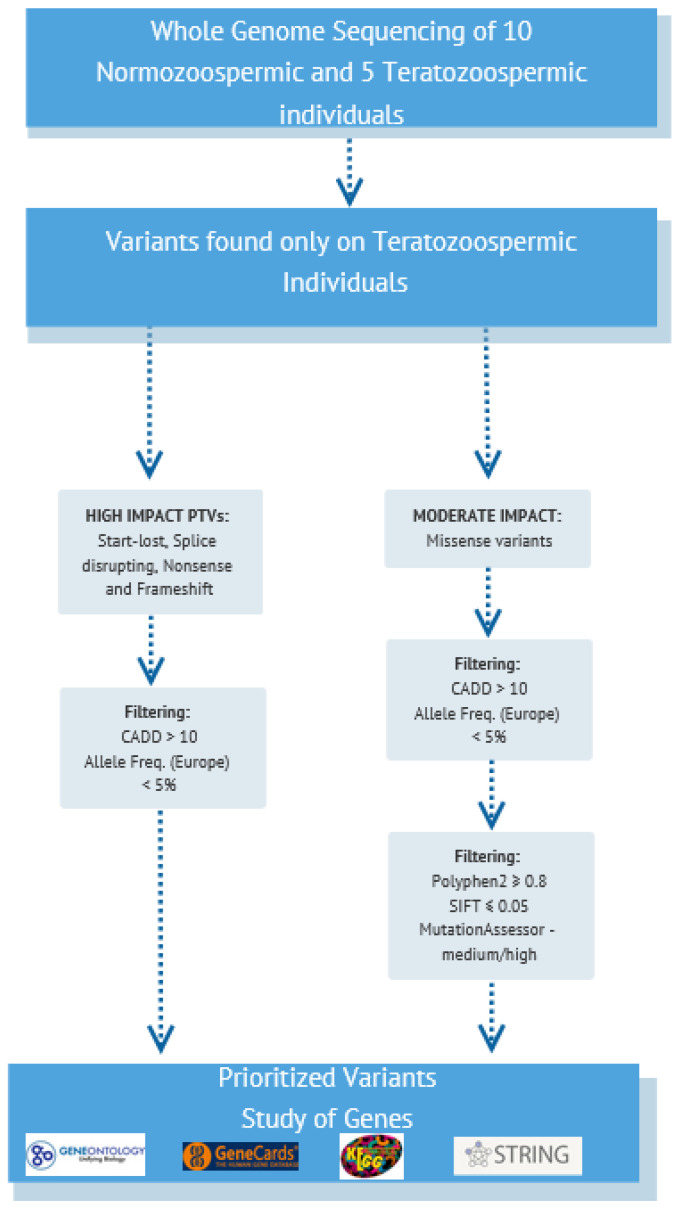
The pipeline used to filter variants found in Greek patients with teratozoospermia. At first, high and moderate impact variants were selected and then, bioinformatics tools were used to assess their impact on protein structure, function, and conservation. Genes on which the prioritized variants were found were further evaluated using several other databases (Gene Ontology; http://geneontology.org/, accessed on 26 June 2022, GeneCards; https://www.genecards.org/, accessed on 26 June 2022, KEGG; https://www.genome.jp/kegg/, accessed on 26 June 2022 and STRING; https://string-db.org/, accessed on 26 June 2022).

In the present study, the variants found only in teratozoospermic individuals were filtered to prioritize those that are more likely to be involved in the occurrence of this specific subtype of male infertility in the Greek population and contribute to the unique genomic profile of Greek patients with teratozoospermia. The corresponding genes that the variants are mapped on were also studied to explore their role. Thus, the prioritization was performed as follows (Figure 1): 

(a): High Impact variants [31]. At first, protein-truncating variants (PTVs) that lead to a truncated protein or its complete absence and thus, can have a severe consequence on protein function [32], as they have been associated with the causing of several diseases according to studies [33], were selected. The PTVs that were prioritized in this study included nonsense single-nucleotide variants (SNVs), frameshift insertions or deletions (indels), splice-disrupting variants, and start-loss SNVs. Common variants with an allele frequency > 0.05 according to data retrieved from the 1000 Genomes Project [25] for the European population as well as from the gnomAD database [26] for Non-Finnish Europeans were excluded. This filter was also applied because the aim was to identify rare variants. According to studies, rare variants can help to shed light on common diseases as they are not found frequently in the population, and thus, there is a greater possibility to be associated with pathogenic phenotypes [13]. It should also be noted that information about allele frequencies was obtained only for the geographical regions mentioned above, as this study is strictly focused on the Greek population. In addition, as an extra filter, a CADD Score > 10 was used to further prioritize the above variants. Any variant with a CADD Score > 10 is considered to be in the top 10% of the human genome’s likely functional and harmful variants.

(b): Moderate Impact Variants [31]. Moderate impact variants, including inframe indels, missense and protein-altering variants, though non-disruptive, have the potential to affect protein’s effectiveness [31]. Of these, we selected to study missense variants as they have been more often associated with complex diseases [34]. In addition, missense variants that can affect protein functionality and which have a low frequency in a population may represent the “missing link” for explaining inheritable diseases [35]. Therefore, missense variants were also chosen and, to assess their effect on protein function, structure, and conservation, bioinformatics prediction tools were used. More specifically, only variants with SIFT Score [28] ≤ 0.05, Polyphen2 Score [27] ≥ 0.8, and with a medium or a high functional impact based on evolutionary conservation as assessed by MutationAssessor [30] were included in the analysis. The criteria regarding CADD score and allele frequency were also applied as in high-impact variants.

In all the above cases, novel variants that are not listed in the existing databases (Ensembl, 1000 Genomes and gnomAD), were also included in this study. 

Furthermore, Gene Ontology (http://www.geneontology.org/GO, accessed on 26 June 2022), KEGG (http://www.genome.jp/kegg/, accessed on 26 June 2022), and GeneCards (https://www.genecards.org/, accessed on 26 June 2022) were used to investigate the role of the genes in which the prioritized variants were found and to identify pathways involved in teratozoospermia. STRING (https://string-db.org/, accessed on 26 June 2022) was also used to study interactions and correlations between proteins encoded by these genes. Genes with more than two prioritized variants were identified, as they may be more likely to be involved in the pathogenesis process and teratozoospermia. 

Finally, Genotype-Tissue Expression (GTEx) database was used in an attempt to explore how the prioritized variants affect gene expression. GTEx Program is a database including information on the relationship between genetic variants and gene expression in multiple human tissues enabling among others the identification of expression quantitative trait loci (eQTLs) [36]. eQTLs are genomic loci that explain at least a fraction of the genetic variance of a gene expression phenotype and thus, can provide valuable information about the role of variants and their effect on phenotype [25]. 

## 3. Results

In brief, in this study, blood samples from normozoospermic and teratozoospermic individuals were used for DNA extraction. After sample preparation, whole-genome sequencing and data analysis were performed, in order to identify unique variants that are present only in teratozoospermic individuals and thus, can contribute to the pathogenic phenotype or have the potential to be used as biomarkers. Then, variant prioritization was performed by applying specific filters and selecting high- and moderate-impact variants that have the greatest possibility to affect protein function and have a role in teratozoospermia according to bioinformatics tools. Therefore, the identified high and moderate impact variants are going to be presented in this section as well as the results of the analyses performed to investigate the role of the genes on which these were found, including GO annotation, KEGG enriched pathways, protein–protein interactions, etc.

### 3.1. Variant Calling and Annotation of WGS Data

After whole-genome sequencing, data analysis was performed. More specifically, the comparison between normozoospermic and teratozoospermic individuals to detect unique variants found only in one of the two groups revealed 617,722 variants specifically observed in teratozoospermic, while 2,342,243 variants were present only in normozoospermic men. The variants were mapped in 34,603 and 22,022 genes and characterized non-coding regions (miRNAs, lncRNAs genes, etc.) in normozoospermic and teratozoospemic males, respectively. 

In the present study, only the variants identified in teratozoospermic individuals were selected for further analysis and prioritization, as the objective was to detect and investigate variants that contribute to the genomic profile of Greek patients with teratozoospermia. Of the 617,722 variants found in teratozoospermic men, 17.29% were mapped in intergenic regions, while the largest proportion (74.15%) was in intronic regions. The remaining variants were mapped in protein-coding regions and, more specifically, 0.62% and 1.07% of all variants were found in the 3’ UTR and 5’ UTR regions, respectively. Moreover, 0.40% of the unique teratozoospermic variants were also synonymous. 

### 3.2. High Impact Variants Identification

In high impact variants, start-lost, nonsense, frameshift, and splice-disrupting variants were included. Thus, 175 variants were characterized first as high impact. Then, further filters were applied to prioritize them. 

More specifically, of the 175 high-impact variants detected, 36 nonsense mutations were found among them. Variants with a CADD score less than 10, as well as variants with allele frequency in the European population greater than 5%, were removed; thus, 20 variants were identified (Table S1) and selected for further pathway enrichment analysis in the next steps. Regarding start-lost mutations, 3 variants specific for teratozoospermic individuals were detected from the WGS analysis, but after the filters referred above were applied, none of them was prioritized. However, 41 frameshift variants were found to be unique in teratozoospermic men, and after prioritization, 22 frameshift mutations (Table S2) were identified. Finally, the highest number of high impact variants were splice-disrupting mutations, since 48 were identified in teratozoospermic men. After filtering, 19 splice-disrupting variants were used for further pathway enrichment analysis (Table S3).

**Figure 2:**
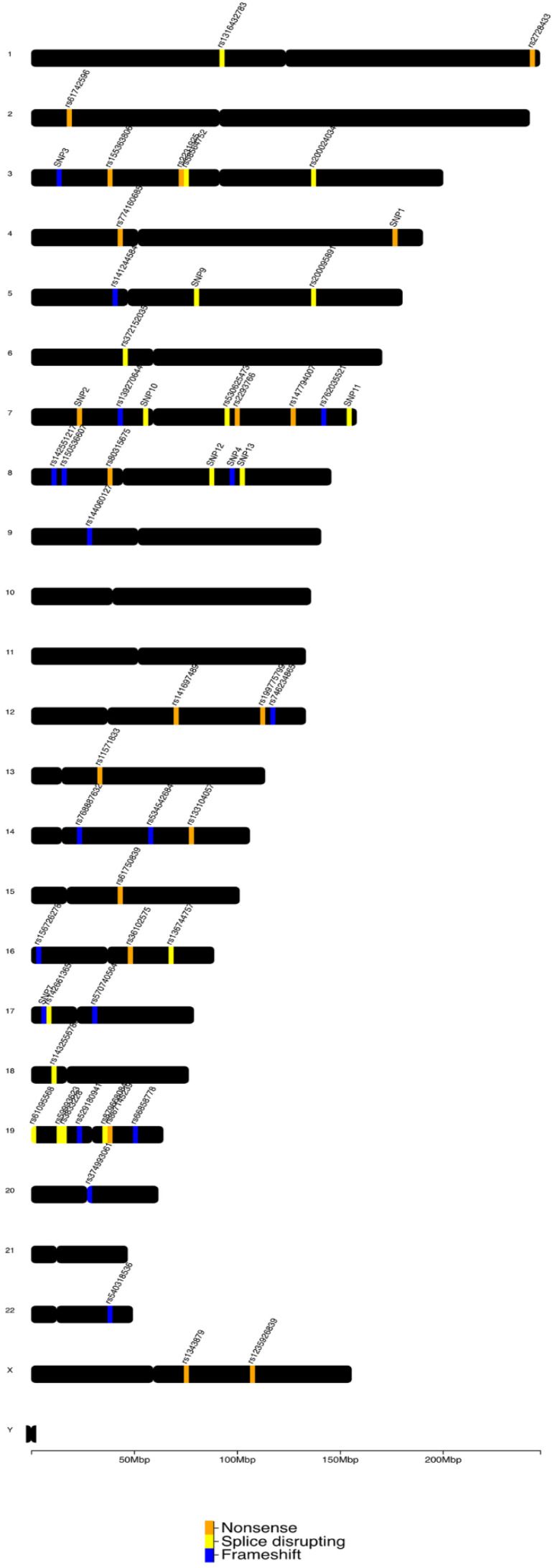
Prioritized high impact variants found on teratozoospermic individuals based on their genomic location (whole-genome representation). Nonsense (orange), splice-disrupting (yellow), and frameshift variants (blue) are distinguished by color as presented. The figure was created with chromoMap [37].

The prioritized variants are presented on whole-genome level in Figure 2.

### 3.3. Moderate Impact Variant Identification

Inframe indels, missense, and protein-altering variants can be characterized as moderate impact because they potentially affect protein function [31]. In the present study, among the variants found only in teratozoospermic individuals, 2141 moderate impact SNVs and indels were identified.

Then, to perform further prioritization, after applying the criteria described in the previous section (CADD Score > 10, Allele frequency (Europe) < 5%, SIFT Score ≤ 0.05, Polyphen2 Score ≥ 0.8, and medium/high impact according to MutationAssessor), 153 missense mutations were identified as the most likely to be associated with teratozoospermia in the Greek population (presented in Table S4). 

### 3.4. Gene Investigation and Enrichment Analysis

After selecting all prioritized variants (total number: 214) as explained above, the next step was to evaluate the role of the genes on which these are found and to explore their potential contribution to male infertility and, particularly, teratozoospermia. More specifically, the full list of genes, as well as their description according to GeneCards [38], is presented in Table S5. 

Based on these results, top genes with more than two prioritized variants from different categories (nonsense, frameshift, splice disrupting, missense) can also be identified as the accumulation of mutations may lead to a greater impact on protein function. These genes are presented in Table 2.

In addition, KEGG enrichment analysis was performed for all the genes identified above. The extracellular matrix (ECM)—receptor interaction pathway was found to be the most gene-enriched and significant pathway, as presented in Table 3. 

**Figure 3:**
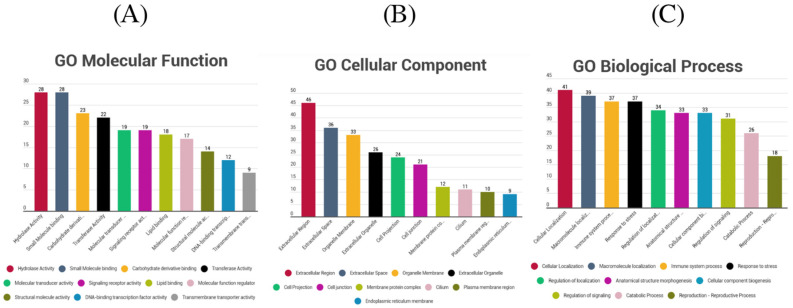
Grouping of genes on which the prioritized variants were found according to GO terms for Molecular Function (**A**), GO terms for Cellular Component (**B**), and GO terms for Biological Process (**C**). The horizontal axis represents the GO terms and the vertical axis the number of genes that were found to be annotated on every GO term.

GO was also used to group genes by functional categories based on GO terms for biological processes, cellular components, and molecular functions. As our gene set was rather small, we performed gene ontology annotation instead of gene ontology enrichment in order to have reliable results and to obtain as much biological information as possible. The most important groups are presented for all three of them in Figure 3A–C. As it is observed, GO Cellular Component analysis revealed many molecules found in the extracellular region, whereas important categories identified in GO Biological Processes were morphogenesis of anatomical structures and reproduction. The most important GO terms regarding the molecular function can be considered the “molecular transducer”, “signaling receptor”, and transferases.

**Figure 4:**
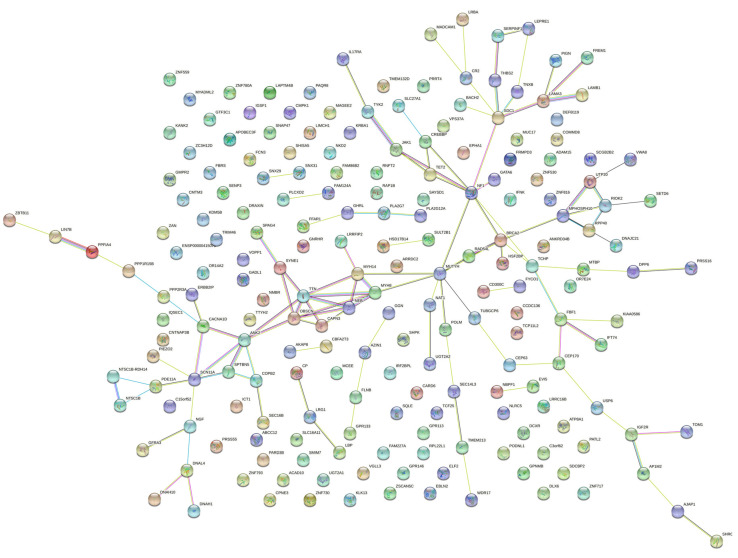
Protein–Protein Interaction (PPI) network for genes on which prioritized variants were found in teratozoospermic men. The network includes 202 nodes and 120 edges. PPI enrichment *p* value is 0.000939.

The STRING database was then used to study the network of the respective proteins of the genes identified above, as protein interaction networks can provide useful information on the molecular basis of complex diseases [39], such as male infertility (Figure 4). The network contained 202 nodes and 120 edges, while coiled-coil proteins were found to be enriched according to Uniprot [40] (Table 4*)*. 

**Figure 5:**
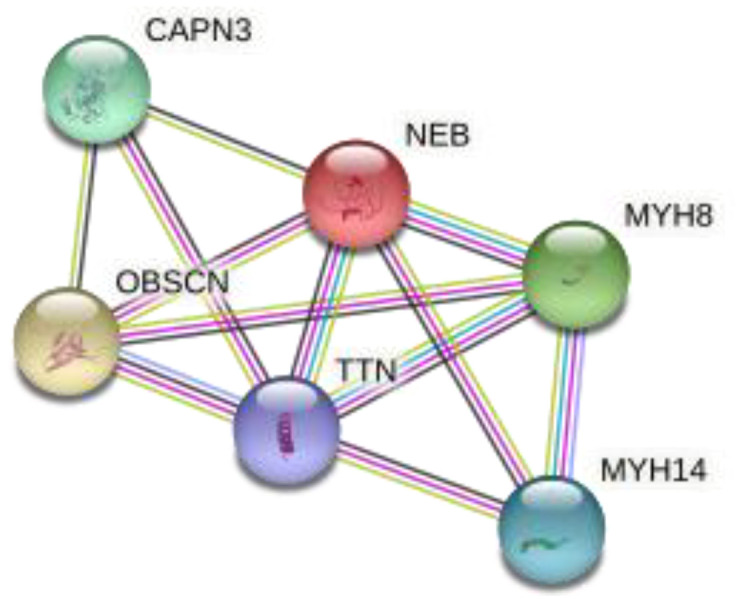
Cluster 1 of the PPI network identified according to MCODE. The cluster consists of 6 nodes and 12 edges.

After that, we also used MCODE [41], a plug-in that is optimized to detect clusters in a network. More specifically, three clusters were calculated according to k-core = 2. Cluster 1 (Figure 5) contained 6 nodes and 12 edges and had the highest score among the identified clusters. The other clusters found on the network are presented in Table 5. 

Finally, to investigate how the prioritized variants can affect gene expression, GTEx was used. It was revealed that some of them alter gene expression, particularly in the testicular tissue. The results are presented in Table 6. 

**Figure 6:**
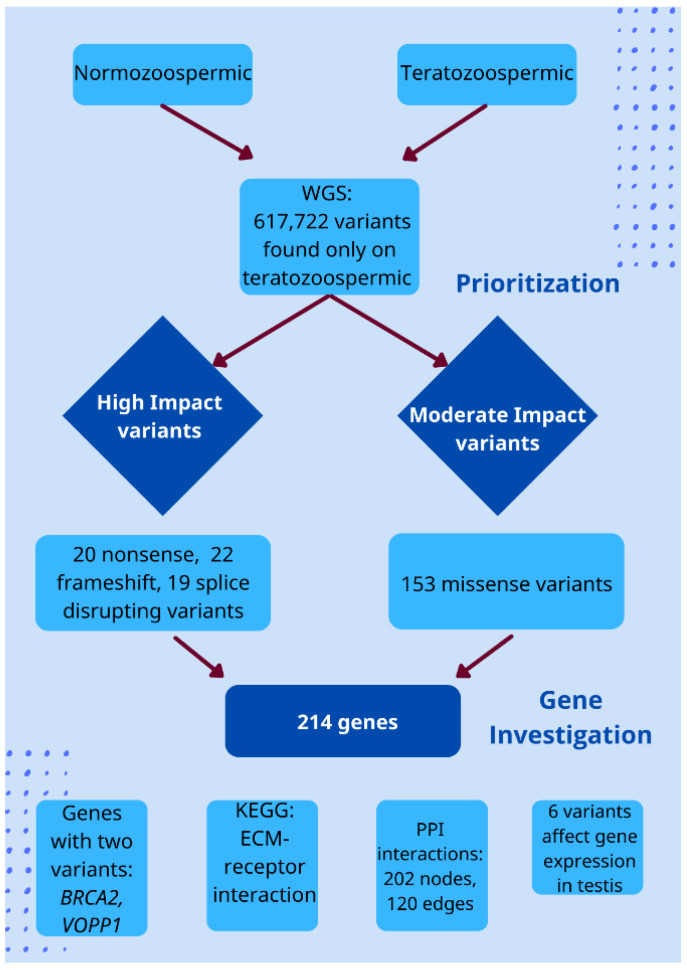
The main results of the present study. As presented, after WGS, 617,722 variants were identified only in teratozoospermic individuals. These were further prioritized to identify those with the greatest possibility to contribute to the pathogenic phenotype. Thus, 20 nonsense, 22 frameshift, and 19 splice-disrupting variants were characterized as high impact variants, being the most likely to affect protein function. A total of 153 missense variants were also identified. Further investigation of the genes on which these were found revealed that two genes carry more than one mutation in teratozoospermic individuals, and most genes play a role in the extracellular matrix–receptor interaction. In addition, many protein interactions were identified and some of the variants can affect gene.

In brief, the main results of the present study are presented in Figure 6.

## 4. Discussion

In the present study, a WGS approach was used to identify and examine variants associated with clinical phenotypes of teratospermia. These may be involved in the molecular basis of teratozoospermia and have the potential to be used to improve the chances of successful ART or the diagnosis of male infertility in specific populations, since significant differences are observed and different genetic factors seem to contribute to infertility among ethnic groups [11,12]. Thus, to fill the gap regarding knowledge on polymorphisms associated with male infertility for Balkan populations, this study focused specifically on the Greek population and teratozoospermia. 

Since next-generation sequencing (NGS) technologies provide massive amounts of data, a thoughtful approach should be followed to filter and select functional variants that are more likely to contribute to the pathogenic phenotype [42]. For this reason, this study focused only on protein-coding variants and after selecting variants found specifically in teratozoospermic individuals, a pipeline was developed that integrated the methodology of Juhari et al. (2021) [43] to prioritize high- and moderate-impact variants involved in teratozoospermia in the Greek population. 

### 4.1. Genes Associated with Male Infertility

In the current study, we discovered high-impact variants specifically found in teratozoospermic men that were mapped in approximately 60 genes (Table S2s). These should be of primary interest and focus in future disease-related studies as they can directly affect protein function [44]. Furthermore, the number of moderate impact or missense variants detected was higher, and these are also of great importance and could provide a list of candidate genes for future studies since they can affect protein structure and functionality leading to diseases [45]. 

Interestingly, many of our candidate genes have been associated in the past with abnormal spermatogenesis, sperm defects, or particular types of male infertility. Among the proteins required for successful fertilization is zonadhesin, involved in the species-specific adhesion of sperm to egg zona pellucida [46], and DCXR, also called “sperm surface protein P34H” [47]. Furthermore, *CREBP* has been suggested to play a role in azoospermia [48] and *FYCO1* was also observed recently to be involved in the regulation of the chromatoid body, which is crucial for spermatogenesis, through autophagy [49].

### 4.2. Genes with Potential Role in Male Infertility and Teratozoospermia

For many of the highlighted genes with prioritized variants, there are indications of their involvement in male infertility, but further research is needed to investigate their exact role. Some of them are centrosomal protein 170 (*CEP170*) [50], gametogenetin (*GGN*) that is a testis-enriched gene [51], ghrelin, and obestatin, identified in human semen and the male reproductive system in general [52,53], Janus Kinase 1 (*JAK1*), found in the midpiece of spermatozoa and activated during capacitation [54,55], the lipopolysaccharide-binding protein that it is found in sperm and tail of spermatozoa [56] and tyrosine-kinase 2 (*TYK2*) that is active in human sperm [57] and plays a role in crucial signaling pathways. *TET2* is also expressed in the cytoplasm of late pachytene spermatocytes of Stage V and there are indications that its expression levels are associated with fertility status and sperm parameters [58]. 

In addition, several of the highlighted genes have been associated with spermatogenesis process or male fertility in other species. These genes should be of primary interest for future studies, as genes involved in spermatogenesis are highly conserved between species [59], and maybe similar molecular patterns are also involved in the presence of male infertility in humans. 

For example, in mice, variants in Serine protease 55 (*Prss55*) [60,61], Centrosomal Protein 63 (*Cep63*) [62], *Ccdc136* [63], *Tom1* [64], *Maps* [65], *Ngf* [66], *Spag4* [67,68], *Pde11a* [69,70], *Atp8a1* [71,72], and *Mtbp* [73] have been associated with deficiencies in reproduction, by affecting among others meiotic recombination, spermatogenesis and sperm structure, acrosome formation, actin-based structures and p53 stability. In cattle, variants in *SEC16B* [74], *VGLL3* [75], and *DEBB119* [76] were also associated with male infertility. Finally, in boar, variants in *ADAM15* [77] and *KDM5B* [78] are associated with fertilization, sperm-egg binding and transcriptional activity in androgen receptors.

This study is also of particular interest because it draws attention to genes that have not been directly associated in the past with teratozoospermia or male infertility but are part of gene families that have members involved in the spermatogenesis process and reproduction in men. More specifically, laminins are important components of testicular basement membranes, and many laminin genes have been characterized as essential for normal testicular function [79]. Variants on laminin subunits have been also identified in the present study (*LAMA3, LAMB1*). Another protein family with a role in a variety of pathological mechanisms is Coiled-Coil Domain-Containing (CCDC) Proteins. Studies suggest that many members of this family, including CCDC42, CCDC9, and CCDC87, are required for the fertilization capacity of males and have been associated with abnormal formation of the sperm flagella [80,81,82]. Coiled-coil proteins were also found to be enriched in the network produced using STRING, thus, *CCDC9B* is a very promising candidate gene for teratozoospermia. *ANK2* may also be a gene of interest, as Ankyrins are a family of proteins linking membrane and submembranous cytoskeletal proteins that play an important role in many cellular functions [83]. However, a specific gene of this family seems to be involved in reproduction as *Ankrd31^−/−^* mice are infertile possibly due to deregulation of the blood-epididymal barrier [84]. Other such genes are *CMTM3*, a member of the chemokine-like factor (CKLF)-like MARVEL transmembrane domain-containing family (CMTM) [85], *AKAP8*, a member of a-kinase anchoring proteins (AKAPs) [86] and *HSD17B14*, as hydroxysteroid (17-beta) dehydrogenase (HSD17B) gene family has a role in steroid hormone biosynthesis and deficiencies in such genes can even lead to sex development disorders [87]. Furthermore, *GATA6* is a candidate gene of importance, since GATA transcription factors play a crucial role in mammalian reproduction [88], whereas KLK13 is part of a protein family involved in semen liquefaction [89] that has the potential to affect sperm quality [90].

Other candidate genes may also be of particular importance for future studies as they interact with proteins involved in reproduction. For example, PPP1R15B has been found to interact with Protein Phosphatase 1 which plays a key role in the spermatogenesis process and has been shown to affect sperm motility [91]. 

### 4.3. The Special Case of BRCA2

It should also be noted that two different variants (missense and nonsense) found only in teratozoospermic individuals were identified in *BRCA2*. This gene has been associated with idiopathic cases of infertility characterized mainly by azoospermia or severe oligozoospermia [92], but in general, there are several studies linking polymorphisms in DNA repair genes with idiopathic infertility in males [93,94]. More interestingly, BRCA2 exhibits a highly evolutionarily conserved interaction with HSF2BP, a testis-specific protein that is essential for mouse spermatogenesis [95]. In the present study, a missense variant characterized as moderate impact was also identified on *HSF2BP* in teratozoospermic individuals suggesting a role of this interaction in teratozoospermia that requires further investigation.

### 4.4. The Role of Genes Associated with Cilia and Flagellum Malformation

Furthermore, the results of the present study are important as they support previous findings associating male infertility with dynein deficiencies [96,97,98,99]. 

Motile cilia and flagella are microtubule-based hair-like structures with a common core component: the axoneme [100]. The axoneme is an evolutionarily conserved structure from protozoa to humans, a fact underlying its significance [101,102]. In sperm flagella, axonemes have a typical “9 + 2” structure with 9 outer doublets of microtubules and a central pair of microtubules [100,103]. Several proteins are also attached to these, including axonemal dyneins classified as outer and inner dynein arms (ODAs and IDAs, respectively) [102,103]. ODAs and IDAs are multiprotein complexes consisting of light, intermediate, and heavy chain proteins [97]. In humans, there are 13 dynein axonemal heavy chain (DNAH) proteins (DNAH1–3, DNAH5–12, DNAH14, and DNAH17) [104] and mutations in many of these genes have been associated with male infertility [96,98,105,106,107]. 

In the present study, several mutations on some of the above genes specifically found in teratozoospermic men were identified. More specifically, a missense variant was identified in *DNAH1*. Mutations in *DNAH1* can cause morphological abnormalities of the sperm flagella and lead to male infertility [103,105,107]. Mutations in *DNAH10,* that exhibits testis-specific expression and causes asthenoteratozoospermia in humans and mice [96], were also identified in teratozoospermic men. Moreover, though Dynein Axonemal Light Chain 4 (*DNAL4*), on which a frameshift variant was found in teratozoospermic men, has not been recorded to be involved in teratozoospermia in humans, researchers observed that a mutation in this gene in boar affected sperm motility and caused midpiece abnormalities [108]. 

Furthermore, variants in teratozoospermic individuals were also identified in genes associated with primary ciliary dyskinesia (PCD), a disease characterized by many symptoms, including male infertility, due to abnormal motile cilia, or celiac disease [103]. These genes affect cilia biogenesis and structure and more specifically, in the present study variants in *FBF1, KIAA0586, ODAD1, GHRL, BACH2,* and *IFT74* were identified. Interestingly, except for its association with primary ciliary dyskinesia, intraflagellar transport protein 74 has been also associated with male infertility as for many years studies highlighted that it was an essential component for the spermatogenesis process in mice [109].

These findings suggest that maybe teratozoospermia is characterized by a set of mutations associated with cilia and flagellum malformation that act accumulative resulting in sperm deficiencies. However, the interesting fact is that these mutations identified here alter sperm morphology but do not have such a strong impact on sperm motility as sperm samples of the present study were characterized only as teratozoospermic and no combinations of male infertility subcategories were included (e.g., asthenoteratozoospermia, etc.). Thus, the identification of these mutations could be useful for distinguishing teratozoospermia from other subcategories of male infertility and providing implications for successful diagnosis. Furthermore, the study of teratozoospermic men harboring mutations that lead to sperm tail abnormalities, as usually are mutations in dyneins, is of importance because studies show that a good sperm nuclear quality in combination with mutations affecting the sperm tail are usually indicators of good embryonic development after intracytoplasmic sperm injection (ICSI) [97]. In particular, mutations in *DNAH1* have been associated in the past with a good pregnancy rate [110], suggesting that further study is required to identify more variants on dynein genes that might contribute to teratozoospermia and have the potential to improve ART outcome or prognosis.

### 4.5. The Role of the Extracellular Matrix in Teratozoospermia

Finally, in the present study, pathway enrichment analysis showed that ECM–receptor interaction was the most significant pathway according to the genes on which prioritized variants were found specifically in Greek teratozoospermic individuals. GO analyses also identified variants in teratozoospermic men on a large number of proteins found in the extracellular region or proteins that are part of the extracellular matrix (ECM), suggesting a crucial role of the ECM in teratozoospermia. 

ECM consists mainly of glycoproteins and polysaccharides but, most importantly, its components interact with a wide range of molecules, including proteases, protease inhibitors, cytokines, etc. [111,112]. Thus, this network of ECM proteins and their partners play a crucial role in the regulation of junction dynamics in testis [111,113]. Junction restructuring is required during germ cell movement in the seminiferous epithelium [112], but as this is a very complex process mediated by several mechanisms [113], it is proposed that protein deficiencies in this network can affect the spermatogenesis process and result in male infertility, as the results of this study indicate. 

Although many studies are highlighting the important role of ECM in the spermatogenesis process, there are no studies directly associating the extracellular matrix with teratozoospermia. However, scientists have previously reported that abnormal basement membrane structures were observed in infertile men with aspermatogenesis [114]. Scientists have also detected in the past that disruption of pathways involved in the ECM and junction dynamics can result in male infertility because germ cells are depleted from the epithelium [112]. Taking into consideration these findings and the results presented here it can be suggested that ECM could play a crucial role in teratozoospermia which was underrated as the abnormal translocation of the germ cells across the seminiferous epithelium can also affect sperm morphology. Therefore, more research is required to identify the molecular mechanism that links ECM with teratozoospermia. 

### 4.6. Prioritized Variants’ Effect on Gene Expression

Investigation of the effect of prioritized variants on gene expression based on the GTEx database for various tissues revealed that some of them affect the mRNA levels of genes on testis tissue (Table 6); more specifically, among them, NT5C1B codes for a protein called autoimmune infertility-related protein that is highly expressed in testis [115]. FBF1 is another interesting gene whose expression seems to be affected by rs113062332. This gene is associated with primary ciliary dyskinesia and in addition, studies in Drosophila prove that is essential for male fertility as RNAi-knockdown flies have impaired sperm flagella and are infertile [116]. GGN is a testis-enriched gene that has also been implicated to have a role in male infertility [51] too. 

### 4.7. Directions for Future Studies 

Future studies aiming at the validation of these findings can be experiments of different types. More specifically, GWAS can be used to investigate if the variants found here are associated with teratozoospermia in large samples of controls–cases. RNA-sequencing experiments can also add valuable information about how the variants affect gene expression and which of the genes identified here are deregulated in teratozoospermia. Finally, functional experiments could also validate the effect of variants on protein function and provide knowledge about their specific impact on the phenotype observed, teratozoospermia. 

Finally, though the variants were filtered and selected to have a low allele frequency, lower than 5%, and it is not likely to be in homozygosity, it should be investigated experimentally, e.g., PCR, if all the variants identified in this paper are found in homozygous or heterozygous state in a large sample of teratozoospermic individuals. More experiments are required to explore if these mutations are dominant or recessive and how they affect protein function potentially using animal models. This information will be extremely valuable, as in dominant mutations that cause male infertility there is a high probability that the use of assisted reproductive technologies will lead to the transfer of this allele to the next generation [117].

## 5. Conclusions

This study is the first comprehensive investigation of the genomic profile of teratozoospermic patients in the Greek population using WGS. It is of importance because it provides a roadmap for future studies, enlisting candidate genes and variants that are associated with teratozoospermia for the first time here and confirming the role of genes that have been studied in the past in male infertility. In particular, the stop-codon variants presented in the present study, that cause termination in protein production, should be further explored as they may shed light on molecular mechanisms and pathways that were previously underrated. Additionally, missense variants detected in teratozoospermic individuals should also be examined. The identification of the important role of the extracellular matrix and the process of cilia and flagellum formation as well as their direct association with teratozoospermia is very promising for future research, too. 

However, the small sample size of the patients recruited is a limitation of the present study. Therefore, validation of these findings in a larger sample could provide more definitive evidence for the role of the variants and the candidate genes in teratozoospermia in the Greek population. Furthermore, assisted reproductive technology (ART) has finally expanded the opportunities for infertile couples, but previous studies have revealed the different outcomes of intracytoplasmic sperm injection (ICSI) for individuals harboring different mutations [11,110]. Thus, further experiments are required to assess and explore the impact of the variants identified in the present study on ICSI outcomes. In this way, by analyzing the genetic profile of a man with teratozoospermia, ICSI would be recommended or not based on the identification of specific mutations as it seems that these affecting only the flagellum structure may have better chances for successful ART outcomes [97]. Finally, it should be noted that synonymous variants and variants in non-coding regions were excluded in this study, but they can also provide useful information and should be investigated for their contribution to teratozoospermia in the future as they can help to fully assess the whole-genome profile of patients.

In conclusion, the present study does not provide conclusive evidence on specific mutations, but the analysis of whole-genome data contributes to our understanding of teratozoospermia by highlighting important pathways and provides the foundation for the improvement of ART and the successful diagnosis or prognosis as a wide spectrum of variants was identified, as well as genes and pathways that had not been explored in the past acting as promising candidates for future research. 

## Figures and Tables

**Table 1:** Demographic information of volunteers selected to participate in this study.

Demographics	Normozoospermic (*n* = 10)	Teratozoospermic (*n* = 5)
**Age**	28–53 Mean = 36	31–49 Mean = 38
**BMI**	19.5–40.4 Mean = 26.97	24.8–33 Mean = 29.24
**Smoking**	30% Not Smoking, 70% Smoking	60% Not Smoking, 40% Smoking
**Alcohol**	100% ≤ 2 drinks/week	80% ≤ 2 drinks/week
**Drug Use**	75% No	80% No
**Nationality**	Greek	Greek

**Table 2:** Genes with more than two prioritized variants of different categories. Variants not found in the 1000 Genomes Project are highlighted because they were also searched in the gnomAD database. Ref; Reference allele, Obs; Observed allele.

Variant	Gene	Ref	Obs	Frequency (Europe)	Type of Mutation
rs11571833	*BRCA2*	A	Τ	0.011	Nonsense
rs766173	*BRCA2*	A	C	0.035	Missense
- (7:55621478-55621503)	*VOPP1*	ACACACACACACACACACACACTCAC	CAC	-	Splice Disrupting
rs201023957	*VOPP1*	C	T	0.000471 (gnomAD)	Missense

**Table 3:** Significant pathways identified after KEGG Enrichment Analysis. FDR; False discovery rate.

KEGG Enrichment Analysis				
Enrichment FDR	Number of Genes	Pathway Genes	Fold Enrichment	Pathways
0.0220	6	88	7.7	ECM–receptor interaction

**Table 4:** Results of the functional enrichment analysis of the network according to Uniprot. The count in network indicates how many proteins in our network are annotated with the term “coiled-coil” of the total number of proteins assigned to the same term. Strength is calculated as log10 (observed/expected) for the number of proteins expected to be annotated with this term in a random network and finally, FDR (false discovery rate) is calculated after multiple testing correction according to Benjamini and Hochberg.

Enriched Annotated Keywords According to Uniprot			
Description term	Count in network	Strength	**FDR**
Coiled Coil	43 of 2139	0.290	0.0114

**Table 5:** Clusters identified in the PPI network according to MCODE and their description based on Genecards.

**Cluster 2**	
RIOK2	RIO Kinase 2
MPHOSPH10	M-Phase Phosphoprotein 10
RPP40	Ribonuclease P/MRP Subunit P40
UTP20	UTP20 Small Subunit Processome Component
**Cluster 3**	
NT5C1B	5′-Nucleotidase, Cytosolic IB
NT5C1B-RDH14	NT5C1B-RDH14 Readthrough
PDE11A	Phosphodiesterase 11A

**Table 6:** Prioritized variants on eQTL regions according to GTEx database.

Variant	Gene	Tissues
rs61742596	*NT5C1B*	Testis
rs61742596	*NT5C1B-RDH14*	Testis
rs61095568	*MADCAM1*	Testis
rs78440807	*UTP20*	Testis
rs113062332	*FBF1*	Testis
rs112138627	*GGN*	Testis
rs77267061	*ZNF559*	Testis

## Data Availability

The sequencing data presented in this study are available through SRA (BioProject ID PRJNA875412, http://www.ncbi.nlm.nih.gov/bioproject/875412 (accessed on 26 June 2022)).

## Supplementary Materials

The following supporting information can be downloaded at: https://www.mdpi.com/article/10.3390/genes13091606/s1, Table S1: Prioritized nonsense variants found in teratozoospermic individuals. Variants not found in the 1000 Genomes Project are highlighted because they were also searched in the gnomAD database. Ref; Reference allele, Obs; Observed allele, Table S2: Prioritized frameshift variants found in teratozoospermic individuals. Variants not found in the 1000 Genomes Project are highlighted because they were also searched in the gnomAD database. Ref; Reference allele, Obs; Observed allele, Table S3: Prioritized splice-disrupting variants found in teratozoospermic individuals. Variants not found in the 1000 Genomes Project are highlighted because they were also searched in the gnomAD database. Ref; Reference allele, Obs; Observed allele, Table S4: Prioritized moderate impact variants found in teratozoospermic individuals; Table S5: Genes on which the prioritized variants (high and moderate impact) were found in teratozoospermic individuals and their description according to GeneCards. The results are presented for every group of variants studied (nonsense, frameshift, splice disrupting, and missense).
