# Supplementary material for: Whole-Genome Profile of Greek Patients with Teratozοοspermia: Identification of Candidate Variants and Genes
Source: Genes (Basel). 2022 Sep 8;13(9):1606. doi: 10.3390/genes13091606 (PMC9498395; doi:10.3390/genes13091606)
Supplement: Supplementary file 1 [file genes-13-01606-s001.zip › Table S1.pdf]

Table S1. Prioritized nonsense variants found in teratozoospermic individuals. Variants not found in the 1000 Genomes Project are highlighted because they were also searched in the gnomAD database. Ref; Reference allele, Obs; Observed allele

Variant	Location	Gene	Ref	Obs	Frequency (Europe)	CADD Score
rs867145239	19:38028548-38028548	*ZNF793*	C	T	0.0000 (gnomAD)	36
rs199775799	12:110340895-110340895	*TCHP*	C	T	0.0002 (gnomAD)	35
rs774160685	4:42461515-42461518	*ATP8A1*	TGT	T	0.0020 (gnomAD)	-
rs2231925	3:73111724-73111724	*EBLN2*	T	G	0.0100	31
rs2293766	7:100371358-100371358	*ZAN*	G	A	0.0100	48
rs11571833	13:32972626-32972626	*BRCA2*	A	T	0.0110	36
rs141697489	12:69050235-69050235	*RAP1B*	G	A	0.0180	10.44
rs1343879	X:75004529-75004529	*MAGEE2*	C	A	0.0260	34
rs36102575	16:48130781-48130781	*ABCC12*	C	T	0.0310	42
rs2728433	1:243328887-243328887	*CEP170*	G	T	0.0410 (gnomAD)	32
rs61750839	15:42162467-42162467	*SPTBN5*	G	A	0.0360	35
rs61742596	2:18766156-18766156	*NT5C1B*	G	T	0.0450	35
rs61742596	2:18766156-18766156	*NT5C1B-RDH14*	G	T	0.0450	35
rs1553638067	3:38941409-38941409	*SCN11A*	C	T	-	32
rs1331040573	14:77493769-77493769	*IRF2BPL*	G	A	-	33
rs1235926839	X:106846442-106846442	*FRMPD3*	C	T	-	34
rs1477940077	7:127999846-127999846	*PRRT4*	C	T	-	36
rs80315675	8:37641780-37641780	*GPR124*	C	G	-	29.2
-	4:177041040-177041040	*WDR17*	G	A	-	-
-	7:23306189-23306189	*GPNMB*	C	T	-	-
