# Supplementary material for: Whole-Genome Profile of Greek Patients with Teratozοοspermia: Identification of Candidate Variants and Genes
Source: Genes (Basel). 2022 Sep 8;13(9):1606. doi: 10.3390/genes13091606 (PMC9498395; doi:10.3390/genes13091606)
Supplement: Supplementary file 1 [file genes-13-01606-s001.zip › Table S2.pdf]

Table S2. Prioritized frameshift variants found in teratozoospermic individuals. Variants not found in the 1000 Genomes Project are highlighted because they were also searched in the gnomAD database. Ref; Reference allele, Obs; Observed allele

Variant	Location	Gene	Ref	Obs	Frequency (Europe)
rs374993061	20:29965207-29965211	*DEFB119*	ACTG	-	0.0000
rs746234865	12:117289581-117289602	*RNFT2*	AGGAA GGAA	AGGAAG GAAGGA AGGAA	0.0000 (gnomAD)
rs570740564	17:29626554-29626555	*NF1*	T	-	0.0000 (gnomAD)
rs2231925	3:73111724-73111724	*EBLN2*	T	G	0.0100
rs768887632	14:22924269-22924270	*TRDJ4*	T	-	0.0001 (gnomAD)
rs762035521	7:143095834-143095850	*EPHA1*	GCCCCC GGGCC CCCGG	GCCCCCG GGCCCCC GGGCCC CCGG	0.0010 (gnomAD genomes)
rs774160685	4:42461515-42461518	*ATP8A1*	TGT	T	0.0020 (gnomAD)
rs139270644	7:44118249-44118251	*POLM*	CT	-	0.0020
rs540318536	22:39176877-39176878	*DNAL4*	C	-	0.0030
rs534542684	14:58899156-58899157	*KIAA058 6*	G	-	0.0070
rs529180941	19:23328751-23328752	*ZNF730*	T	TT	0.0070
rs66858778	19:49619102-49619103	*LIN7B*	G	-	0.0000 (gnomAD)
rs150536607	8:17137320-17137322	*VPS37A*	TA	-	0.0230
rs144060127	9:27524364-27524371	*IFNK*	TGTTTG T	TGTTTGT TTGT	0.0300
rs141244584	5:40841571-40841573	*CARD6*	TT	-	0.0320
rs142551217	8:10411512-10411515	*PRSS55*	GGG	GG	0.0360
-	3:12942848-12942850	*IQSEC1*	GG	G	-
-	8:98788168-98788183	*LAPTM4 B*	GGGCT CCAGG CGAGG	GGGCTCC AGGCGA GGAGGG CTCCAGG CGAGG	-
-	14:22591984-22591990	*TRAV26-1*	CCCCCC	CCCCCCC	-
rs1567262786	16:3779217-3779219	*CREBBP*	CC	CCC	-
-	16:89952390-89952453	*TCF25*	TCTCTG CGGCT GCCCTT CTCTGC GGCTG CCCTTC TCTGCG GCTGCC CTTCTC TGCGG CTGCCC	TCTCTGC GGCTGCC CGTCTCT GCGGCTG CCCTTCT CTGCGGC TGCCCTT CTCTGCG GCTGCCC TTCTCTG CGGCTGC CC	-
-	17:6945529-6945548	*SLC16A1 1*	CACAG CCCCA	CA	-

-	17:79995492-79995493	*DCXR*	GCCCAGTCAT	TGT	-
