# Supplementary material for: Whole-Genome Profile of Greek Patients with Teratozοοspermia: Identification of Candidate Variants and Genes
Source: Genes (Basel). 2022 Sep 8;13(9):1606. doi: 10.3390/genes13091606 (PMC9498395; doi:10.3390/genes13091606)
Supplement: Supplementary file 1 [file genes-13-01606-s001.zip › Table S3.pdf]

Table S3. Prioritized splice-disrupting variants found in teratozoospermic individuals. Variants not found in the 1000 Genomes Project are highlighted because they were also searched in the gnomAD database. Ref; Reference allele, Obs; Observed allele

Variant	Location	Gene	Ref	Obs	Frequency (Europe)	CADD Score
rs1316432783	1:92979088-92979126	*EVI5*	TATATATATATATATATATATATATATATATATATGTA	TA	0.0005 (gnomAD genomes)	-
rs58564752	3:75834065-75834065	*ZNF717*	C	T	0.0290	12.78
rs200024034	3:135825050-135825056	*PPP2R3A*	CAATCA	CAATCAATCA	0.0260	-
-	5:79864769-79864772	*ANKRD34B*	AGAG	GAGAGAGAGAGAGTGAG	-	-
rs200095891	5:137588969-137588972	*GFRA3*	CCC	CCCCC	0.0200	-
rs372152035	6:46690519-46690519	*PLA2G7*	C	A	0.0088 (gnomAD exomes)	34
-	7:55621478-55621503	*VOPP1*	ACACACACACACACACACACACTCAC	CAC	-	-
rs530625473	7:96635363-96635411	*DLX6*	GCAGCAGCAGCAGCAGCAGCAACA	GCAGCAGCAGCAGCAGCAGCAACAGCAGCAGCAGCAGCAGCAGCA	0.0010	-
-	7:154378874-154378874	*DPP6*	A	G	-	-
-	8:87536928-87536930	*CPNE3*	TTG	TTTTTGTG	-	-
-	8:103862219-103862234	*AZIN1*	TTACACACCTGCTACA	TACA	-	-
rs1367447570	16:66638633-66638633	*CMTM3*	G	A	-	22.1
rs536045893	17:5071419-5071419	*USP6*	G	A	0.0000	28.9
rs1426613652	17:7475188-7475237	*SENP3*	ATATATATATATATATATATATATATATAT	ATATATA	-	-

			ATATATATAAAAATATATA			
rs1432556786	18:10666808-10666811	*PIEZO2*	AGC	-	-	-
rs61095568	19:490040-490040	*MADCAM1*	G	A	0.0100	12.30
rs59993623	19:14050681-14050704	*PODNL1*	AATAATAATAATAATAATAATAA	AATAATAATAATAATAATAATAATAATAA	0.0001 (gnomAD)	-
rs3833228	19:16770761-16770766	*SMIM7*	CTACT	CTACTACT	0.0130	-
rs879668084	19:35099589-35099590	*SCGB2B2*	A	ATATATATATACA	0.0000 (gnomAD)	-
