# Supplementary material for: Whole-Genome Profile of Greek Patients with Teratozοοspermia: Identification of Candidate Variants and Genes
Source: Genes (Basel). 2022 Sep 8;13(9):1606. doi: 10.3390/genes13091606 (PMC9498395; doi:10.3390/genes13091606)
Supplement: Supplementary file 1 [file genes-13-01606-s001.zip › Table S5.pdf]

Table S5: Genes on which the prioritized variants (high and moderate impact) were found in teratozoospermic individuals and their description according to GeneCards. The results are presented for every group of variants studied (nonsense, frameshift, splice disrupting, and missense)

**Nonsense Variants**			
Gene	***Description***	***Gene***	***Description***
ZNF793	Zinc Finger Protein 793	*CEP170*	Centrosomal Protein 170
TCHP	Trichoplein Keratin Filament Binding	*SPTBN5*	Spectrin Beta, Non-Erythrocytic 5
ATP8A1	ATPase Phospholipid Transporting 8A1	*NT5C1B*	5'-Nucleotidase, Cytosolic IB
EBLN2	Endogenous Bornavirus Like Nucleoprotein 2	*NT5C1B-RDH14*	NT5C1B-RDH14 Readthrough
ZAN	Zonadhesin	*SCN11A*	Sodium Voltage-Gated Channel Alpha Subunit 11
BRCA2	BRCA2 DNA Repair Associated	*IRF2BPL*	Interferon Regulatory Factor 2 Binding Protein Like
RAP1B	RAP1B, Member of RAS Oncogene Family	*FRMPD3*	FERM And PDZ Domain Containing 3
MAGEE2	MAGE Family Member E2	*PRRT4*	Proline Rich Transmembrane Protein 4
ABCC12	ATP Binding Cassette Subfamily C Member 12	*GPR124/ADGRA2*	Adhesion G Protein-Coupled Receptor A2
WDR17	WD Repeat Domain 17	*GPNMB*	Glycoprotein Nmb
**Frameshift Variants**			
DEFB119	Defensin Beta 119	*TRDJ4*	T Cell Receptor Delta Joining 4
RNFT2	Ring Finger Protein, Transmembrane 2	*EPHA1*	EPH Receptor A1
NF1	Neurofibromin 1	*ATP8A1*	ATPase Phospholipid Transporting 8A1
POLM	DNA Polymerase Mu	*DNAL4*	Dynein Axonemal Light Chain 4
KIAA0586	KIAA0586	*ZNF730*	Zinc Finger Protein 730
DCXR	Dicarbonyl And L-Xylulose Reductase	*VPS37A*	VPS37A Subunit Of ESCRT-I
IFNK	Interferon Kappa	*CARD6*	Caspase Recruitment Domain Family Member 6
PRSS55	Serine Protease 55	*IQSEC1*	IQ Motif and Sec7 Domain ArfGEF 1
LAPTM4B	Lysosomal Protein Transmembrane 4 Beta	*TRAV26-1*	T Cell Receptor Alpha Variable 26-1
CREBBP	CREB Binding Protein	*TCF25*	Transcription Factor 25
SLC16A11	Solute Carrier Family 16 Member 11	*LIN7B*	Lin-7 Homolog B, Crumbs Cell Polarity Complex Component
**Splice Disrupting Variants**			

EVI5	Ecotropic Viral Integration Site 5	*PPP2R3A*	Protein Phosphatase 2 Regulatory Subunit B''Alpha
ZNF717	Zinc Finger Protein 717	*ANKRD34B*	Ankyrin Repeat Domain 34B
GFRA3	GDNF Family Receptor Alpha 3	*PLA2G7*	Phospholipase A2 Group VII
VOPP1	VOPP1 WW Domain Binding Protein	*DLX6*	Distal-Less Homeobox 6
DPP6	Dipeptidyl Peptidase Like 6	*CPNE3*	Copine 3
AZIN1	Antizyme Inhibitor 1	*CMTM3*	CKLF Like MARVEL Transmembrane Domain Containing 3
USP6	Ubiquitin Specific Peptidase 6	*SENP3*	SUMO Specific Peptidase 3
PIEZO2	Piezo Type Mechanosensitive Ion Channel Component 2	*MADCAM1*	Mucosal Vascular Addressin Cell Adhesion Molecule 1
PODNL1	Podocan Like 1	*SMIM7*	Small Integral Membrane Protein 7
SCGB2B2	Secretoglobin Family 2B Member 2		
**Missense Variants**			
UTP20	UTP20 Small Subunit Processome Component	*TCP11L2*	T-Complex 11 Like 2
ACAD10	Acyl-CoA Dehydrogenase Family Member 10	*DNAH10*	Dynein Axonemal Heavy Chain 10
TMEM132D	Transmembrane Protein 132D	*GPR133/ ADGRD1*	Adhesion G Protein-Coupled Receptor D1
BRCA2	BRCA2 DNA Repair Associated	*VWA8*	Von Willebrand Factor A Domain Containing 8
FAM124A	Family With Sequence Similarity 124 Member A	*TRAV8-4*	T Cell Receptor Alpha Variable 8-4
TRAV24	T Cell Receptor Alpha Variable 24	*LRRC16B/ CARMIL3*	Capping Protein Regulator and Myosin 1 Linker 3
GMPR2	Guanosine Monophosphate Reductase 2	*C15orf52/ CCDC9B*	Coiled-Coil Domain Containing 9B
CAPN3	Calpain 3	*PATL2*	PAT1 Homolog 2
SNX29	Sorting Nexin 29	*GTF3C1*	General Transcription Factor IIIC Subunit 1
FBRS	Fibrosin	*NLRC5*	NLR Family CARD Domain Containing 5
SETD6	SET Domain Containing 6, Protein Lysine Methyltransferase	*CBFA2T3*	CBFA2/RUNX1 Partner Transcriptional Co-Repressor 3
MYH8	Myosin Heavy Chain 8	*SERPINF1*	Serpin Family F Member 1
SHPK	Sedoheptulokinase	*TTYH2*	Tweety Family Member 2

CD300C	Immunoglobulin Superfamily Member 16	*ICT1/ MRPL58*	Mitochondrial Ribosomal Protein L58
FBF1	Fas Binding Factor 1	*MYADML2*	Myeloid Associated Differentiation Marker Like 2
GATA6	GATA Binding Protein 6	*LAMA3*	Laminin Subunit Alpha 3
PIGN	Phosphatidylinositol Glycan Anchor Biosynthesis Class N	*TYK2*	Tyrosine Kinase 2
AP1M2	Adaptor Related Protein Complex 1 Subunit Mu 2	*KANK2*	KN Motif and Ankyrin Repeat Domains 2
AKAP8	A-Kinase Anchoring Protein 8	*SLC27A1*	Solute Carrier Family 27 Member 1
ARRDC2	Arrestin Domain Containing 2	*FFAR1*	Free Fatty Acid Receptor 1
GGN	Gametogenetin	*ZNF780A*	Zinc Finger Protein 780A
LRG1	Leucine Rich Alpha-2-Glycoprotein 1	*CCDC114/ODAD1*	Outer Dynein Arm Docking Complex Subunit 1
SULT2B1	Sulfotransferase Family 2B Member 1	*HSD17B14*	Hydroxysteroid 17-Beta Dehydrogenase 14
MYH14	Myosin Heavy Chain 14	*KLK13*	Kallikrein Related Peptidase 13
ZNF816	Zinc Finger Protein 816	*ZSCAN5C*	Zinc Finger and SCAN Domain Containing 5C
ZNF530	Zinc Finger Protein 530	*OR7E24*	Olfactory Receptor Family 7 Subfamily E Member 24
ZNF559	Zinc Finger Protein 559	*ZNF559-ZNF177*	ZNF559-ZNF177 Readthrough
NGF	Nerve Growth Factor	*DRAXIN*	Dorsal Inhibitory Axon Guidance Protein
ADAM15	ADAM Metallopeptidase Domain 15	*TRIM46*	Tripartite Motif Containing 46
KDM5B	Lysine Demethylase 5B	*PPFIA4*	PTPRF Interacting Protein Alpha 4
NBPF1	NBPF Member 1	*SEC16B*	SEC16 Homolog B, Endoplasmic Reticulum Export Factor
PPP1R15B	Protein Phosphatase 1 Regulatory Subunit 15B	*CR2*	Complement C3d Receptor 2
SNAP47	Synaptosome Associated Protein 47	*OBSCN*	Obscurin, Cytoskeletal Calmodulin And Titin-Interacting RhoGEF
OR14A2	Olfactory Receptor Family 14 Subfamily A Member 2	*FCN3*	Ficolin 3
LEPRE1/ P3H1	Prolyl 3-Hydroxylase 1	*MUTYH*	MutY DNA Glycosylase
RAD54L	RAD54 Like	*CMPK1*	Cytidine/Uridine Monophosphate Kinase 1

AJAP1	Adherens Junctions Associated Protein 1	*JAK1*	Janus Kinase 1
SDCBP2	Syndecan Binding Protein 2	*SPAG4*	Sperm Associated Antigen 4
LBP	Lipopolysaccharide Binding Protein	*HSF2BP*	Heat Shock Transcription Factor 2 Binding Protein
IL17RA	Interleukin 17 Receptor A	*IGLV9-49*	Immunoglobulin Lambda Variable 9-49
SEC14L3	SEC14 Like Lipid Binding 3	*TOM1*	Target Of Myb1 Membrane Trafficking Protein
FAM227A	Family With Sequence Similarity 227 Member A	*APOBEC3F*	Apolipoprotein B MRNA Editing Enzyme Catalytic Subunit 3F
TUBGCP6	Tubulin Gamma Complex Associated Protein 6	*NEB*	Nebulin
PDE11A	Phosphodiesterase 11A	*TTN*	Titin
SDC1	Syndecan 1	*PARD3B*	Par-3 Family Cell Polarity Regulator Beta
GPR113/ ADGRF3	Adhesion G Protein-Coupled Receptor F3	*MCEE*	Methylmalonyl-CoA Epimerase
MPHOSPH10	M-Phase Phosphoprotein 10	*ZBTB11*	Zinc Finger and BTB Domain Containing 11
GHRL	Ghrelin And Obestatin Prepropeptide	*PLCXD2*	Phosphatidylinositol Specific Phospholipase C X Domain Containing 2
CEP63	Centrosomal Protein 63	*COPB2*	COPI Coat Complex Subunit Beta 2
CP	Ceruloplasmin	*RPL22L1*	Ribosomal Protein L22 Like 1
GADL1	Glutamate Decarboxylase Like 1	*LRRFIP2*	LRR Binding FLII Interacting Protein 2
FYCO1	FYVE And Coiled-Coil Domain Autophagy Adaptor 1	*SHISA5*	Shisa Family Member 5
C3orf62	Chromosome 3 Open Reading Frame 62	*DNAH1*	Dynein Axonemal Heavy Chain 1
CACNA1D	Calcium Voltage-Gated Channel Subunit Alpha1 D	*FLNB*	Filamin B
VGLL3	Vestigial Like Family Member 3	*TET2*	Tet Methylcytosine Dioxygenase 2
PLA2G12A	Phospholipase A2 Group XIIA	*ANK2*	Ankyrin 2
ELF2	E74 Like ETS Transcription Factor 2	*LRBA*	LPS Responsive Beige-Like Anchor Protein
LIMCH1	LIM And Calponin Homology Domains 1	*COMMD8*	COMM Domain Containing 8
GNRHR	Gonadotropin Releasing Hormone Receptor	*UGT2A1*	UDP Glucuronosyltransferase Family 2 Member A1 Complex Locus

UGT2A2	UDP Glucuronosyltransferase Family 2 Member A2	*NKD2*	NKD Inhibitor of WNT Signaling Pathway 2
DNAJC21	DnaJ Heat Shock Protein Family (Hsp40) Member C21	*ERBB2IP/ ERBIN*	Erbb2 Interacting Protein
RIOK2	RIO Kinase 2	*NMBR*	Neuromedin B Receptor
ZC3H12D	Zinc Finger CCCH-Type Containing 12D	*SYNE1*	Spectrin Repeat Containing Nuclear Envelope Protein 1
IGF2R	Insulin Like Growth Factor 2 Receptor	*THBS2*	Thrombospondin 2
PRSS16	Serine Protease 16	*SAYSD1*	SAYSVFN Motif Domain Containing 1
RPP40	Ribonuclease P/MRP Subunit P40	*PAQR8*	Progestin And AdipoQ Receptor Family Member 8
BACH2	BTB Domain And CNC Homolog 2	*MUC17*	Mucin 17, Cell Surface Associated
LAMB1	Laminin Subunit Beta 1	*GPR146*	G Protein-Coupled Receptor 146
CCDC136	Coiled-Coil Domain Containing 136	*TMEM213*	Transmembrane Protein 213
KRBA1	KRAB-A Domain Containing 1	*VOPP1*	VOPP1 WW Domain Binding Protein
SNX31	Sorting Nexin 31	*MTBP*	MDM2 Binding Protein
FAM86B2	Family With Sequence Similarity 86 Member B2	*SQLE*	Squalene Epoxidase
C8orf17	Chromosome 8 Putative Open Reading Frame 17	*NAT1*	N-Acetyltransferase 1
FREM1	FRAS1 Related Extracellular Matrix 1	*IFT74*	Intraflagellar Transport 74
CNTNAP3B	Contactin Associated Protein Family Member 3B	*TNXB*	Tenascin XB
IGSF1	Immunoglobulin Superfamily Member 1	*SHROOM2*	Shroom Family Member 2
SLC9B1P1	Solute Carrier Family 9 Member B1 Pseudogene 1		
